# 1-(3-Chloro­phen­yl)-3-(2,6-dichloro­benzo­yl)thio­urea

**DOI:** 10.1107/S1600536808043444

**Published:** 2009-01-08

**Authors:** M. Khawar Rauf, Michael Bolte, Abdur Rauf

**Affiliations:** a Department of Chemistry, Quaid-i-Azam University, Islamabad 45320, Pakistan; b Institut für Anorganische Chemie, J. W. Goethe-Universität Frankfurt, Max-von-Laue-Strasse 7, 60438 Frankfurt/Main, Germany; c Department of Chemistry, Islamia University of Bahawalpur, Pakistan

## Abstract

The structure of the title compound, C_14_H_9_Cl_3_N_2_OS, is composed of discrete mol­ecules with bond lengths and angles quite typical for thio­urea compounds of this class. The plane containing the thio­carbonyl and carbonyl groups subtends dihedral angles of 48.19 (3) and 87.51 (3)° with the planes formed by the 3-chloro and 2,6-dichloro­phenyl rings, respectively; the dihedral angle between the two benzene ring planes is 45.32 (3)°. An intra­molecular N—H⋯O hydrogen bond stabilizes the mol­ecular conformation and the mol­ecules form inter­molecular N—H⋯S and N—H⋯O hydrogen bonds, generating a sheet along the *a* axis.

## Related literature

For related structures, see: Khawar Rauf *et al.*, (2006*a* ▶,*b* ▶; 2007 ▶). For a description of the Cambridge structural Database, see: Allen (2002 ▶).
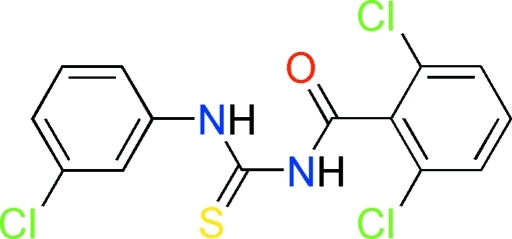


## Experimental

### 

#### Crystal data

C_14_H_9_Cl_3_N_2_OS
*M* *_r_* = 359.64
Monoclinic, 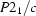

*a* = 10.6589 (5) Å
*b* = 11.2114 (5) Å
*c* = 13.2919 (6) Å
β = 99.942 (3)°
*V* = 1564.55 (12) Å^3^
*Z* = 4
Mo *K*α radiation
μ = 0.72 mm^−1^
*T* = 173 (2) K
0.47 × 0.47 × 0.45 mm

#### Data collection

Stoe IPDS-II two-circle diffractometer
Absorption correction: multi-scan (*MULABS*; Spek, 2003 ▶; Blessing, 1995 ▶) *T*_min_ = 0.729, *T*_max_ = 0.739
39690 measured reflections
5066 independent reflections
4674 reflections with *I* > 2σ(*I*)
*R*_int_ = 0.046

#### Refinement

*R*[*F* ^2^ > 2σ(*F* ^2^)] = 0.037
*wR*(*F* ^2^) = 0.094
*S* = 1.06
5066 reflections
199 parameters
H atoms treated by a mixture of independent and constrained refinement
Δρ_max_ = 0.65 e Å^−3^
Δρ_min_ = −0.66 e Å^−3^

### 

Data collection: *X-AREA* (Stoe & Cie, 2001 ▶); cell refinement: *X-AREA*; data reduction: *X-AREA*; program(s) used to solve structure: *SHELXS97* (Sheldrick, 2008 ▶); program(s) used to refine structure: *SHELXL97* (Sheldrick, 2008 ▶); molecular graphics: *PLATON* (Spek, 2003 ▶) and *XP* in *SHELXTL-Plus* (Sheldrick, 2008 ▶); software used to prepare material for publication: *PLATON* and *SHELXL97*.

## Supplementary Material

Crystal structure: contains datablocks I, global. DOI: 10.1107/S1600536808043444/pv2127sup1.cif

Structure factors: contains datablocks I. DOI: 10.1107/S1600536808043444/pv2127Isup2.hkl

Additional supplementary materials:  crystallographic information; 3D view; checkCIF report

## Figures and Tables

**Table 1:** Hydrogen-bond geometry (Å, °)

*D*—H⋯*A*	*D*—H	H⋯*A*	*D*⋯*A*	*D*—H⋯*A*
N2—H2⋯O1	0.82 (2)	2.07 (2)	2.7190 (13)	136.0 (18)
N2—H2⋯O1^i^	0.82 (2)	2.37 (2)	3.0749 (14)	145.5 (18)
N1—H1⋯S1^ii^	0.86 (2)	2.47 (2)	3.2974 (10)	163.6 (18)

Symmetry codes: (i) 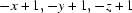; (ii) 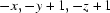.

**Fig. 1.**
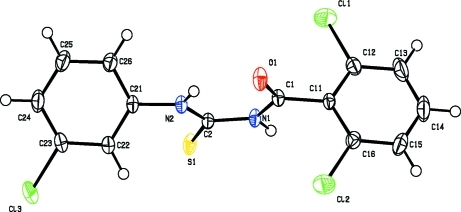
Molecular structure of (I) showing atom numbering scheme. Displacement ellipsoids are drawn at the 50% probability level.

**Fig. 2.**
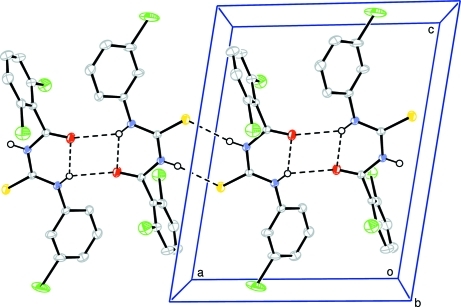
A view of the unit cell of (I) showing hydrogen bonds as dashed lines. H atoms not involved in hydrogen bonding have been omitted for clarity.

## supplementary crystallographic information

### Comment

The background to this study has been set out in our previous work on the structural chemistry of N,N'-disubstituted thioureas (Khawar Rauf et al., 2006a, 2007). Herein, as a continuation of these studies, the structure of the title compound, (I), is described.

In the structure of the title compound (Fig. 1), bond lengths and bond angles can be regarded as typical for N,N'-disubstituted thiourea compounds as found in the Cambridge Structural Database v5.28 (Allen, 2002) and some related structures (Khawar Rauf et al., 2006b). The molecule exists in the thione form with typical thiourea C—S and C—O bonds, as well as shortened C—N bonds. The thiocarbonyl and carbonyl groups are almost coplanar. The molecule features an intramolecular N—H···O hydrogen bond in the crystal structure. The molecules lying about inversions centers associate via N—H···S intermolecular hydrogen bonds to form dimers on one side and a similar association via N—H···O hydrogen bonding on the other side thus result in a sheet of molecules of (I) along the a<ι>-axis (Table 1; Fig. 2).

### Experimental

Freshly prepared and steam distillated 2,6-dichlorobenzoyl isothiocyanate (2.32 g, 10 mmol) was stirred in acetone (30 ml) for 20 minutes. Neat 3-chloroaniline (1.27 g, 10 mmol) was then added and the resulting mixture was stirred for 1 h. The reaction mixture was then poured into acidified (pH 4) water (approx. 300 ml) and stirred well. The solid product was separated, washed with deionized water and purified by recrystallization from methanol/1,1-dichloromethane (1:10 *v/v*) to give fine crystals of (I), with an overall yield of 85%.

### Refinement

Hydrogen atoms bonded to C were included in calculated positions and refined as riding on their parent C atom with C—H = 0.95 Å and *U*_iso_(H) = 1.2*U*_eq_(C). The H atoms bonded to N were freely refined.

### Crystal data

**Table:**

C_14_H_9_Cl_3_N_2_OS	*F*(000) = 728
*M**_r_* = 359.64	*D*_x_ = 1.527 Mg m^−^^3^
Monoclinic, *P*2_1_/*c*	Mo *K*α radiation, λ = 0.71073 Å
Hall symbol: -P 2ybc	Cell parameters from 37695 reflections
*a* = 10.6589 (5) Å	θ = 3.7–31.2°
*b* = 11.2114 (5) Å	µ = 0.72 mm^−^^1^
*c* = 13.2919 (6) Å	*T* = 173 K
β = 99.942 (3)°	Block, colourless
*V* = 1564.55 (12) Å^3^	0.47 × 0.47 × 0.45 mm
*Z* = 4	

### Data collection

**Table:**

Stoe IPDS-II two-circle diffractometer	5066 independent reflections
Radiation source: fine-focus sealed tube	4674 reflections with *I* > 2σ(*I*)
graphite	*R*_int_ = 0.046
ω scans	θ_max_ = 31.3°, θ_min_ = 3.6°
Absorption correction: multi-scan (*MULABS*; Spek, 2003; Blessing, 1995)	*h* = −15→15
*T*_min_ = 0.729, *T*_max_ = 0.739	*k* = −16→16
39690 measured reflections	*l* = −19→19

### Refinement

**Table:**

Refinement on *F*^2^	Secondary atom site location: difference Fourier map
Least-squares matrix: full	Hydrogen site location: inferred from neighbouring sites
*R*[*F*^2^ > 2σ(*F*^2^)] = 0.037	H atoms treated by a mixture of independent and constrained refinement
*wR*(*F*^2^) = 0.094	*w* = 1/[σ^2^(*F*_o_^2^) + (0.0404*P*)^2^ + 0.9365*P*] where *P* = (*F*_o_^2^ + 2*F*_c_^2^)/3
*S* = 1.06	(Δ/σ)_max_ = 0.001
5066 reflections	Δρ_max_ = 0.65 e Å^−^^3^
199 parameters	Δρ_min_ = −0.66 e Å^−^^3^
0 restraints	Extinction correction: *SHELXL97* (Sheldrick, 2008), Fc^*^=kFc[1+0.001xFc^2^λ^3^/sin(2θ)]^-1/4^
Primary atom site location: structure-invariant direct methods	Extinction coefficient: 0.0111 (12)

### Special details

**Table:**

Geometry. All e.s.d.'s (except the e.s.d. in the dihedral angle between two l.s. planes) are estimated using the full covariance matrix. The cell e.s.d.'s are taken into account individually in the estimation of e.s.d.'s in distances, angles and torsion angles; correlations between e.s.d.'s in cell parameters are only used when they are defined by crystal symmetry. An approximate (isotropic) treatment of cell e.s.d.'s is used for estimating e.s.d.'s involving l.s. planes.
Refinement. Refinement of *F*^2^ against ALL reflections. The weighted *R*-factor *wR* and goodness of fit *S* are based on *F*^2^, conventional *R*-factors *R* are based on *F*, with *F* set to zero for negative *F*^2^. The threshold expression of *F*^2^ > σ(*F*^2^) is used only for calculating *R*-factors(gt) *etc*. and is not relevant to the choice of reflections for refinement. *R*-factors based on *F*^2^ are statistically about twice as large as those based on *F*, and *R*- factors based on ALL data will be even larger.

### Fractional atomic coordinates and isotropic or equivalent isotropic displacement parameters (Å^2^)

**Table:**

	*x*	*y*	*z*	*U*_iso_*/*U*_eq_	
S1	0.09625 (3)	0.60027 (3)	0.62517 (2)	0.02474 (9)	
Cl1	0.19574 (4)	0.61905 (4)	0.23352 (3)	0.03999 (11)	
Cl2	0.22323 (4)	0.18815 (4)	0.41908 (3)	0.03969 (11)	
Cl3	0.33122 (7)	0.61484 (7)	0.99688 (3)	0.0735 (2)	
C1	0.27158 (11)	0.45474 (11)	0.41547 (8)	0.0182 (2)	
O1	0.38737 (8)	0.46303 (10)	0.43625 (7)	0.0285 (2)	
N1	0.19003 (9)	0.49493 (10)	0.47738 (8)	0.01915 (19)	
H1	0.1105 (19)	0.4841 (18)	0.4559 (15)	0.030 (5)*	
C2	0.21866 (10)	0.55197 (10)	0.57210 (8)	0.0174 (2)	
N2	0.34238 (9)	0.56439 (10)	0.61243 (8)	0.01918 (19)	
H2	0.394 (2)	0.5442 (19)	0.5770 (15)	0.032 (5)*	
C11	0.20320 (10)	0.39859 (11)	0.31773 (8)	0.0179 (2)	
C12	0.16489 (13)	0.46736 (13)	0.23029 (10)	0.0251 (2)	
C13	0.09985 (17)	0.41613 (18)	0.14040 (11)	0.0394 (4)	
H13	0.0745	0.4639	0.0814	0.047*	
C14	0.07264 (17)	0.29526 (19)	0.13789 (12)	0.0421 (4)	
H14	0.0275	0.2606	0.0770	0.051*	
C15	0.11043 (15)	0.22420 (15)	0.22299 (12)	0.0337 (3)	
H15	0.0923	0.1412	0.2208	0.040*	
C16	0.17558 (12)	0.27683 (12)	0.31200 (10)	0.0232 (2)	
C21	0.39003 (11)	0.62132 (11)	0.70818 (9)	0.0195 (2)	
C22	0.34464 (15)	0.58955 (14)	0.79662 (10)	0.0289 (3)	
H22	0.2822	0.5288	0.7953	0.035*	
C23	0.39279 (16)	0.64880 (16)	0.88702 (10)	0.0340 (3)	
C24	0.48645 (15)	0.73570 (15)	0.89168 (11)	0.0332 (3)	
H24	0.5180	0.7752	0.9541	0.040*	
C25	0.53305 (14)	0.76367 (15)	0.80313 (11)	0.0318 (3)	
H25	0.5984	0.8218	0.8053	0.038*	
C26	0.48479 (12)	0.70721 (13)	0.71106 (10)	0.0246 (2)	
H26	0.5164	0.7273	0.6507	0.030*	

### Atomic displacement parameters (Å^2^)

**Table:**

	*U*^11^	*U*^22^	*U*^33^	*U*^12^	*U*^13^	*U*^23^
S1	0.01676 (13)	0.03383 (17)	0.02412 (15)	−0.00159 (11)	0.00495 (10)	−0.01452 (12)
Cl1	0.0485 (2)	0.02974 (18)	0.0415 (2)	−0.00112 (15)	0.00718 (16)	0.00931 (14)
Cl2	0.0507 (2)	0.02793 (17)	0.0402 (2)	0.00109 (15)	0.00724 (16)	0.00754 (14)
Cl3	0.1095 (5)	0.0945 (5)	0.02015 (18)	−0.0585 (4)	0.0216 (2)	−0.0165 (2)
C1	0.0160 (4)	0.0228 (5)	0.0156 (4)	0.0010 (4)	0.0018 (4)	−0.0044 (4)
O1	0.0144 (4)	0.0461 (6)	0.0246 (4)	0.0004 (4)	0.0023 (3)	−0.0134 (4)
N1	0.0133 (4)	0.0275 (5)	0.0163 (4)	−0.0008 (3)	0.0016 (3)	−0.0081 (4)
C2	0.0171 (5)	0.0197 (5)	0.0152 (4)	−0.0009 (4)	0.0018 (4)	−0.0037 (4)
N2	0.0153 (4)	0.0266 (5)	0.0152 (4)	−0.0008 (4)	0.0015 (3)	−0.0061 (4)
C11	0.0160 (4)	0.0231 (5)	0.0147 (4)	−0.0007 (4)	0.0030 (4)	−0.0051 (4)
C12	0.0247 (6)	0.0317 (6)	0.0185 (5)	−0.0009 (5)	0.0023 (4)	0.0000 (4)
C13	0.0408 (8)	0.0571 (10)	0.0173 (6)	−0.0037 (7)	−0.0037 (5)	−0.0014 (6)
C14	0.0391 (8)	0.0612 (11)	0.0243 (6)	−0.0127 (8)	0.0002 (6)	−0.0189 (7)
C15	0.0315 (7)	0.0355 (7)	0.0350 (7)	−0.0098 (6)	0.0080 (5)	−0.0185 (6)
C16	0.0225 (5)	0.0246 (6)	0.0236 (5)	−0.0020 (4)	0.0070 (4)	−0.0059 (4)
C21	0.0185 (5)	0.0235 (5)	0.0151 (4)	−0.0003 (4)	−0.0005 (4)	−0.0049 (4)
C22	0.0358 (7)	0.0330 (7)	0.0168 (5)	−0.0123 (5)	0.0015 (5)	−0.0041 (5)
C23	0.0446 (8)	0.0414 (8)	0.0156 (5)	−0.0123 (7)	0.0041 (5)	−0.0064 (5)
C24	0.0341 (7)	0.0410 (8)	0.0222 (6)	−0.0079 (6)	−0.0016 (5)	−0.0123 (5)
C25	0.0265 (6)	0.0382 (8)	0.0300 (6)	−0.0108 (5)	0.0027 (5)	−0.0129 (6)
C26	0.0206 (5)	0.0305 (6)	0.0229 (5)	−0.0044 (5)	0.0038 (4)	−0.0076 (5)

### Geometric parameters (Å, °)

**Table:**

S1—C2	1.6766 (12)	C13—H13	0.9500
Cl1—C12	1.7313 (15)	C14—C15	1.385 (3)
Cl2—C16	1.7386 (14)	C14—H14	0.9500
Cl3—C23	1.7433 (15)	C15—C16	1.3951 (18)
C1—O1	1.2209 (14)	C15—H15	0.9500
C1—N1	1.3721 (14)	C21—C26	1.3912 (18)
C1—C11	1.5128 (15)	C21—C22	1.3925 (18)
N1—C2	1.3980 (14)	C22—C23	1.3912 (18)
N1—H1	0.86 (2)	C22—H22	0.9500
C2—N2	1.3423 (14)	C23—C24	1.389 (2)
N2—C21	1.4360 (14)	C24—C25	1.390 (2)
N2—H2	0.82 (2)	C24—H24	0.9500
C11—C12	1.3957 (17)	C25—C26	1.3947 (17)
C11—C16	1.3957 (17)	C25—H25	0.9500
C12—C13	1.3971 (19)	C26—H26	0.9500
C13—C14	1.385 (3)		
			
O1—C1—N1	124.03 (10)	C14—C15—C16	118.77 (14)
O1—C1—C11	122.98 (10)	C14—C15—H15	120.6
N1—C1—C11	112.99 (9)	C16—C15—H15	120.6
C1—N1—C2	128.90 (10)	C15—C16—C11	121.96 (13)
C1—N1—H1	116.6 (13)	C15—C16—Cl2	118.99 (11)
C2—N1—H1	114.5 (13)	C11—C16—Cl2	119.05 (9)
N2—C2—N1	116.98 (10)	C26—C21—C22	120.68 (11)
N2—C2—S1	125.50 (9)	C26—C21—N2	118.43 (11)
N1—C2—S1	117.52 (8)	C22—C21—N2	120.88 (11)
C2—N2—C21	124.95 (10)	C23—C22—C21	118.48 (13)
C2—N2—H2	117.2 (14)	C23—C22—H22	120.8
C21—N2—H2	117.5 (14)	C21—C22—H22	120.8
C12—C11—C16	117.79 (11)	C24—C23—C22	121.91 (13)
C12—C11—C1	120.90 (11)	C24—C23—Cl3	119.18 (10)
C16—C11—C1	121.31 (11)	C22—C23—Cl3	118.89 (12)
C11—C12—C13	121.04 (14)	C23—C24—C25	118.67 (12)
C11—C12—Cl1	119.67 (10)	C23—C24—H24	120.7
C13—C12—Cl1	119.28 (12)	C25—C24—H24	120.7
C14—C13—C12	119.59 (15)	C24—C25—C26	120.60 (13)
C14—C13—H13	120.2	C24—C25—H25	119.7
C12—C13—H13	120.2	C26—C25—H25	119.7
C13—C14—C15	120.85 (13)	C21—C26—C25	119.62 (12)
C13—C14—H14	119.6	C21—C26—H26	120.2
C15—C14—H14	119.6	C25—C26—H26	120.2
			
O1—C1—N1—C2	0.6 (2)	C14—C15—C16—C11	0.2 (2)
C11—C1—N1—C2	−179.35 (12)	C14—C15—C16—Cl2	179.98 (12)
C1—N1—C2—N2	−4.11 (19)	C12—C11—C16—C15	−0.86 (18)
C1—N1—C2—S1	175.27 (11)	C1—C11—C16—C15	178.74 (12)
N1—C2—N2—C21	179.40 (11)	C12—C11—C16—Cl2	179.39 (9)
S1—C2—N2—C21	0.07 (18)	C1—C11—C16—Cl2	−1.01 (16)
O1—C1—C11—C12	−90.46 (16)	C2—N2—C21—C26	−130.55 (14)
N1—C1—C11—C12	89.48 (14)	C2—N2—C21—C22	50.86 (18)
O1—C1—C11—C16	89.95 (16)	C26—C21—C22—C23	2.4 (2)
N1—C1—C11—C16	−90.11 (14)	N2—C21—C22—C23	−179.07 (14)
C16—C11—C12—C13	0.68 (19)	C21—C22—C23—C24	−1.7 (3)
C1—C11—C12—C13	−178.93 (13)	C21—C22—C23—Cl3	176.96 (13)
C16—C11—C12—Cl1	179.53 (10)	C22—C23—C24—C25	−0.1 (3)
C1—C11—C12—Cl1	−0.07 (16)	Cl3—C23—C24—C25	−178.76 (14)
C11—C12—C13—C14	0.1 (2)	C23—C24—C25—C26	1.3 (3)
Cl1—C12—C13—C14	−178.73 (14)	C22—C21—C26—C25	−1.2 (2)
C12—C13—C14—C15	−0.8 (3)	N2—C21—C26—C25	−179.82 (13)
C13—C14—C15—C16	0.6 (2)	C24—C25—C26—C21	−0.6 (2)

### Hydrogen-bond geometry (Å, °)

**Table:**

*D*—H···*A*	*D*—H	H···*A*	*D*···*A*	*D*—H···*A*
N2—H2···O1	0.82 (2)	2.07 (2)	2.7190 (13)	136.0 (18)
N2—H2···O1^i^	0.82 (2)	2.37 (2)	3.0749 (14)	145.5 (18)
N1—H1···S1^ii^	0.86 (2)	2.47 (2)	3.2974 (10)	163.6 (18)

Symmetry codes: (i) −*x*+1, −*y*+1, −*z*+1; (ii) −*x*, −*y*+1, −*z*+1.
